# Consequences of COVID-19 for geriatric patients during a pandemic

**DOI:** 10.1038/s41598-024-84379-z

**Published:** 2025-01-24

**Authors:** Ludwig Wemhöner, Charlotte Brandts, Hannah Dinse, Eva-Maria Skoda, Sarah Jansen, Martin Teufel, Hana Rohn, Richard Dodel

**Affiliations:** 1 Department of Geriatric Medicine, University Duisburg-Essen, Essen, Germany; 2 Department of Infectious Diseases, West German Centre of Infectious Diseases, University Medicine Essen University Hospital Essen, University Duisburg-Essen, Essen, Germany; 3 Clinic for Psychosomatic Medicine and Psychotherapy, LVR-University Hospital, University of Duisburg-Essen, Essen, Germany; 4 Center for Translational Neuro- and Behavioral Sciences (C-TNBS), University of Duisburg-Essen, Essen, Germany; 5 Chair of Geriatric Medicine, University Duisburg-Essen, Virchowstrasse 171, 45356 Essen, Germany

**Keywords:** Covid-19, Pandemic, Infection, Mortality, Comprehensive geriatric treatment, Diseases, Medical research

## Abstract

To investigate the outcomes of geriatric COVID-19 patients in a German academic setting during the pandemic. This study included 468 consecutive geriatric patients (≥ 70 years) who tested positive for SARS-CoV-2 and were treated at the University of Duisburg-Essen from 2/2020 to 3/2021. 74 patients were transferred to a geriatric hospital and a 12-month follow-up (prospective study) was performed in 51 patients. Clinical assessments evaluated depression (GDS), apathy (AES), cognitive status (MMST), mobility (TUG), health status (EQ-5D-5 L), and daily living activities (Barthel Index). Demographic and clinical data were also analyzed. Results showed that the mortality in this vulnerable group was 52% (*n* = 209). Long-term survival was higher in patients who received comprehensive geriatric treatment (74.3% vs. 51.8%). The duration of inpatient stay at the primary hospital was 13.3 ± 3.6 days, with 28.8% (*n* = 135) requiring intensive care. At the 12-month mark more patients with geriatric treatment lived in nursing homes. Barthel-Index/Timed-Up-and-Go-Test/MMST/AES/GDS, and EQ-5D-5 L indicated worse outcomes in the group who received geriatric treatment. Specialized geriatric care may improve survival in geriatric COVID-19 patients despite decreased long-term outcomes. Further research, including international studies like NAPKON, are encouraged to confirm these findings and explore potential interventions for improved outcomes in this vulnerable population.

## Introduction

The emergence of viral pathogens, like SARS-CoV-2, poses a continuous challenge. As of September 2023, there have been 770 million reported cases and 6.9 million deaths^1–3^.

Demographic changes, such as an aging population, present new challenges to medicine. In Germany, the population aged over 65 years reached 22% in 2021 and is expected to increase to 25 million by 2040^4,5^. This relevant group of our society is underrepresented with minimal attention, especially during the pandemic. Elderly individuals face severe courses of SARS-CoV-2 infections^6,7^ with high hospitalization, ICU admission, and death rates. In total, 45% of hospitalized patients, 53% of ICU admissions and 80% of deaths^9^ were among adults 65 years and older, with the highest percentage of severe outcomes occurring in those aged 85 years and above^8^. In a recent review, the case fatality rate increased from 3% in individuals younger than 50 years of age to 19% in those aged 50 years and older^10^. Data from Italy showed that the fatality rose from 0.4% in the group aged ≤ 40 years to 12.8% in the 70–79 age group and further to 20.2% in those aged ≥ 80 years^11^.

Advanced age, along with the comorbidities that often accompany it, such as frailty, sarcopenia, and dementia, may play a significant role in influencing treatment outcome, mortality rates, and recovery from COVID-19 disease^12–15^. For instance, every point increase in the clinical frailty scale has been associated with a 12% rise in mortality^14^, and having two or more comorbidities doubles the probability experiencing a severe outcome^16^. Furthermore, factors such as social distancing, disruption in regular activities, and reduced contacts with medical professionals can contribute to the development of depressive symptoms, decreased resilience, cognitive decline, and an increased risk of sarcopenia among older individuals^17,18^.

Vaccination against SARS-CoV-2 is effective in preventing severe outcomes, with reduced infection (OR: 0.38) and death rates (OR: 0.16) among older populations^19^. Despite a slightly decreased vaccination response in the elderly, vaccination remains effective. (83.8% compared to 89.1%)^20^.

There is limited research on the outcome and survival of geriatric patients following COVID-19 infection. This study aims to investigate the journey of geriatric individuals during the pandemic and their outcomes one year later in a German academic setting.

## Materials and methods

### Study design

The study included all consecutive geriatric patients who tested positive for SARS-CoV-2 and received treatment either in the Department of Infectious Diseases or in the Department of Geriatric Medicine at the University of Essen during the period from February 2020 to March 2021. The inclusion criteria for the study were: persons with age 70 years or older and a positive COVID-19 RT-PCR test shortly before or during the stay at the University Hospital Essen.

**Fig. 1:**
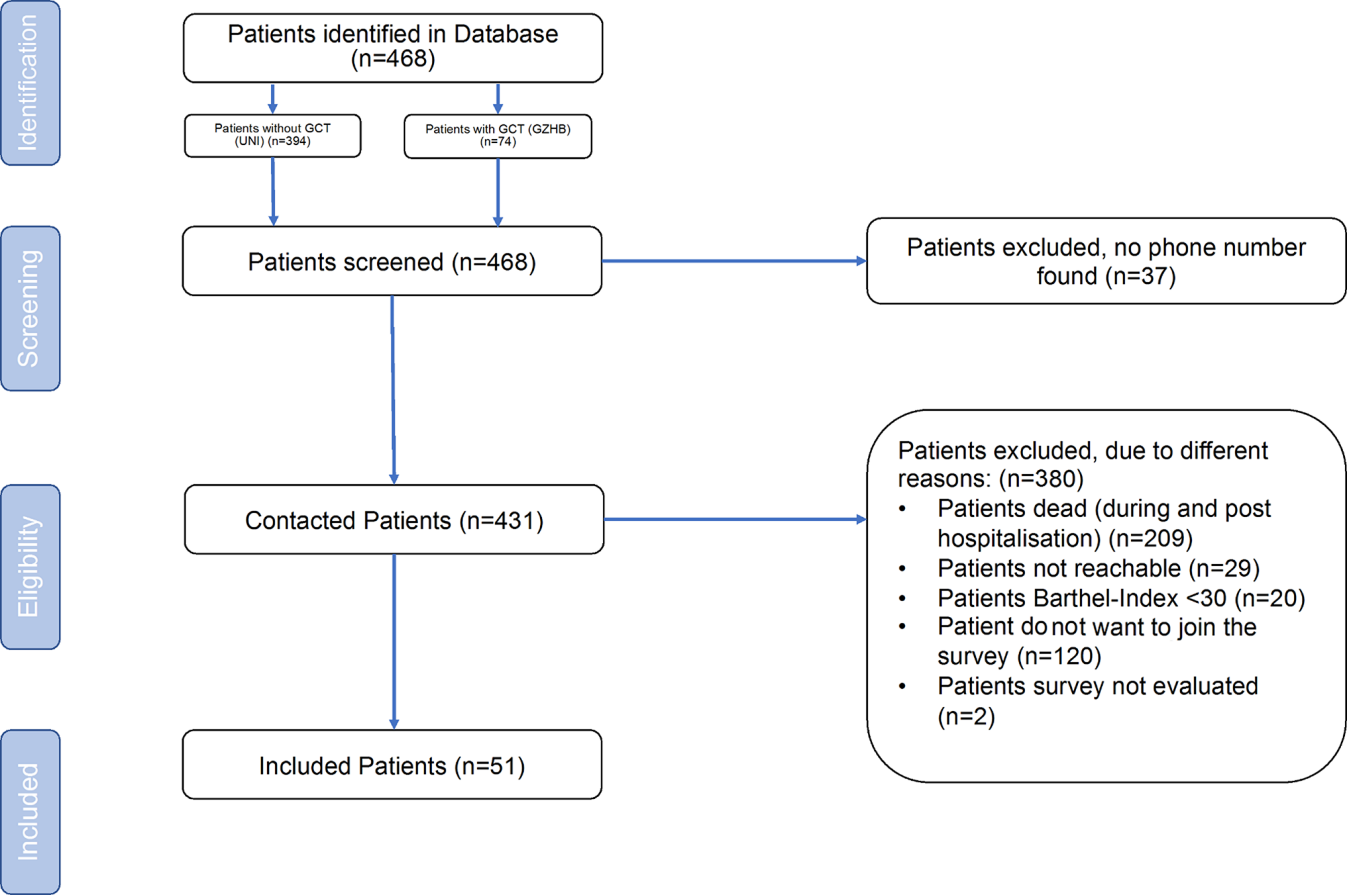
Algorithm and reasons for patient enrollment or exclusion. A total of 468 potentially eligible patients were identified. A total of 51 patients were included on the 12-month evaluation.

As of the cut-off date of August 28th, 2021, the University Hospital Essen had provided treatment to a total of 1,596 inpatients with COVID-19. Among these, 468 inpatients (29.3%) met the criteria for “geriatric patients” (age ≥ 70 y with comorbidities). Seventy-four patients were subsequently transferred to the Department of Geriatrics for early complex geriatric rehabilitation, while 394 remained at the University Hospital for the entire duration of their treatment (Fig. 1). The selection criteria for a transfer to the Department of Geriatrics depended on the one hand on individual functional impairment, limitations in mobility and neurological impairments (e.g. delirium). On the other hand, it depended on the individual rehabilitative potential and prognosis. Patients who previously come from home were stratified as follows due to limited resources: (1) Patients with an uncomplicated course. These patients had only minor restrictions as a result of the acute COVID-19 disease. In most cases, we tried to discharge them. (2) Patients with a more severe course. These patients were significantly more severely affected by physical and cognitive limitations as a result of the disease(s). These patients were presented to the Department of Geriatrics - unless the patient themselves or their relatives had expressed other wishes.

Early complex geriatric rehabilitation (“geriatrische frührehabilitative Komplextherapie”) is a special treatment procedure in which intensive rehabilitation measures are carried out at an early stage in addition to the diagnostic and therapeutic measures required by a hospital due to an acute illness. Its efficacy has been evaluated in a number of studies. The aim is to preserve and/or restore the disease-related functional deficits in order to guarantee mobility and activities of daily living (ADL) in addition to curative treatment. Structural prerequisites are the comprehensive geriatric assessment and the existence of an interdisciplinary geriatric team.

This study includes both a retrospective and a prospective part. In the prospective part, the geriatric persons were clinically examined at the 12 month period after discharge. We were able to identify 431 persons out of the 468 patients (for 37 patients no phone contact/address was available); 29 persons did not react back to repeated phone calls or letter correspondence, resulting in 402 persons (see Fig. 1). 193 persons were alive at the 12 month mark. Of these, 51 agreed to participate in the study (*n* = 35 treated exclusively at the Department of Infectious Diseases and *n* = 16 treated at the Geriatric Department). Reasons for not participating in the study were due to severely poor physical conditions hindering clinic or at home visits (Barthel Index < 30 points, *n* = 20), inability to communicate (*n* = 2) or lack of interest in participating in the study (*n* = 120).

The clinical information of the inpatients, which account for the retrospective part of the study, was extracted from the clinical files of the Department of Infectious Diseases or from the Department of Geriatric Medicine at the University of Essen. It was used to analyze mortality, duration of stay, and the need of intensive care treatment.

The prospective clinical assessment at the 12 months mark included a thorough medical history review, a general and neurologic examination as well as a number of clinical outcome measures as outlined below. This assessment was used to further obtain a prospective view on the clinical situation of geriatric patients following COVID-19 infection.

The study protocol was approved by the Ethics Committee of the Medical Faculty of the University Duisburg-Essen and research was performed in accordance with relevant guidelines and regulations. All participants or their legal guardians, who took part at the 12 month evaluation, gave informed consent. The study was performed in accordance with the Declaration of Helsinki (https://www.wma.net/policies-post/wma-declaration-of-helsinki/).

### Clinical outcome measures

#### Activities of daily living

The Barthel index is used to assess disability and monitor changes in daily-life activities^21^. This index comprises ten different subitems that are rated on a scale of 15/10/5/0 points: feeding, bathing, grooming, dressing, bowel-control, bladder-control, toilet-use, transfer (bed to chair), mobility (on level surfaces), and stairs. A maximum score of 100 points indicates full autonomy, while a score of 0 signifies complete dependence on another person^22^. Cut-off scores: 0–20 indicates “total” dependency, 21–60 indicates “severe” dependency, 61–90 indicates “moderate” dependency, and 91–99 indicates “slight” dependency^23^.

#### Patients’ health status

Patients’ health status was measured using the EQ-5D-5L, which assesses five dimensions: mobility, self-care, usual activities, pain/discomfort, and anxiety/depression^24^. Each dimension offers five possible responses: Level 1: no problem; Level 2: slight problems; Level 3: moderate problems; Level 4: severe problems; Level 5: extreme problems. The second part of the EuroQol is a visual analogue scale (EQ-VAS), which allows patients to rate their current health status on a scale ranging from 0 to 100^25^.

#### Depression

The 15-item GDS “Geriatric Depression Scale” was chosen to assess depression in the elderly population^26^. This scale encompasses the affective symptoms of depression, such as sadness, apathy and crying, as well as cognitive domains as thoughts of hopelessness, guilt and worthlessness, which are represented in 15 self-rating questions^27^. The cut-off values range from 0 to 5 points (indicating inconspicuous findings), 5–10 points (suggestive of mild to moderate depression) and 10–15 points (indicative of major depression)^28^.

#### Apathy

The Apathy Evaluation Scale (AES) was utilized to measure apathy, which is characterized as the lack of motivation. It is reflected at the levels of cognition, (observable) behaviour and emotion/affect^29^. The scale contains eighteen items, with each item rated on a scale of one (does not apply at all) to four points (applies very much). The cut-off score for apathy is 39–41^30^. There are three different versions of the AES: AES-S self-rated, AES-C clinician-rated and AES-I informant-rated. For our study, we employed the self-rated version^31^.

#### Cognitive status

The cognitive screening test “Mini-Mental-State-Test” (MMST) was used^32^. The maximum that can be achieved is 30 points, which means no cognitive impairment and the minimum is 0 points^33^. The results were interpreted according to the German guidelines for Dementia (Maier et al. 2016): MMST 20 to 26 points: mild dementia, MMST 10 to 19 points: moderate/moderate severe dementia, MMST below 10 points: severe dementia.

#### Mobility

The Timed up and Go Test” (TUG) evaluates basic mobility skills, such as strength, balance, and agility. This test measures the time taken to rise from a chair, walk three meters, turn and return to a seated position^34^. A TUG-time below 10 s indicates that daily mobility is unrestricted with no significant limitations in daily activities. Ten to 19 s point to mild mobility impairment, usually without relevance to daily activities. 20–29 s potentially concerns to relevant functional mobility impairment that requires further investigation. The results above 30 s are indicators for severe mobility impairment, including pronounced limitations in mobility^35^. The TUG-Test was assessed once within 48 h of admission to and on discharge from the Geriatric hospital. In addition, TUG-Test was performed at the 12 month evaluation.

#### Statistical analysis

Data entry was performed using an ACCESS data bank (Microsoft, Redmond, Washington/USA). Statistical analysis was done with IBM SPSS Statistics version 26.0.0.0. (IBM, Armonk, New York/USA). Descriptive statistics and percentages were calculated. For all the numeric variables we assessed mean, standard deviation, median, range and number of cases. For all the other variables we assessed the number of cases and percentages. Comparisons between two independent groups were accomplished with nonparametric Mann–Whitney U-test statistics. A *p*-value < 0.05 was determined as significant.

## Results

The clinical characteristics of the cohort can be found in Table 1. The mean duration of inpatient stay at the University Hospital Essen for the entire cohort was 13.3 ± 13.6 days. A total of 135 out of 468 patients (28.8%) were in need of supportive intensive care treatment. 124 patients (26.5%) died during the hospital stay; 85 persons (18.2%) passed away within the first year after discharge (March 2022). Thus, a total of 209 of the 402 patients reached (52%) died, while 193 patients (48.0%) survived one year after hospital discharge (Table 1; Fig. 1).

### Care at the geriatric hospital

A total of 74 patients were admitted to the Geriatric Department for early complex geriatric rehabilitation (Table 2). The mean age among these patients was 81.8 ± 6.6 years, with 29 (39.2%) males and 45 (60.8%) female patients. The mean inpatient stay was 18.6 ± 10.3 days, with men staying for 16.9 ± 9.7 days and women for 19.6 ± 10.6 days.

Three patients (4.1%) died in the hospital, 16 (21.6%) died during the first year after discharge from the hospital and 48 (71,6%) survived the first year after discharge (Table 1); seven of the patients in this group were not reachable; thus, the survival status is uncertain.

We assessed the Barthel index at both admission to the hospital and discharge. The mean Barthel index increased from 39 ± 23.7 to 53.3 ± 31.3 points (*p* = 0.007). When stratified by sex, the male group improved from 32.8 ± 24.5 to 42.1 ± 31.9 points (*p* = 0.267), and the female group improved from 42.5 ± 22.8 to 59.3 ± 29.7 points (*p* = 0.004).

The TUG test (mean duration) at the time of admission to the hospital was 22.3 ± 13.1 s, and at discharge, it was 21.0 ± 12.8 s. Interestingly, male performance worsened from admission to discharge (27.5 ± 25.7 vs. 33.0 ± 22.7 s) (*p* = 0.730), while female performance improved from 20.6 ± 6.5 s to 18.0 ± 7 s (*p* = 0.273).

Regarding the GDS, the mean value at the time of admission was 3.0 ± 2.7 points. Notably, males had a slightly higher score of 3.4 ± 3.3 points compared to females, who had a mean score of 2.9 ± 2.4 (*p* = 0.497) (Table 2).

The MMST at admission revealed a mean value of 20.1 ± 7.7 points, with males scoring 15.9 ± 8.8 points and females scoring 22.3 ± 6.1 points.

**Table 1:** Information of the patient cohort (*n* = 468).

Variable	Whole cohort (*n* = 468; x̅±SD; median, range)	Patients no geriatric care (*n* = 394; $\overline {{\text{x}}}$±SD; median, range)	Patients with geriatric care (*n* = 74; $\overline {{\text{x}}}$ ±SD; median, range)	*p*-value
Age	80.7 ± 6.6 (80.3, 68–99)	80.5 ± 6.6 (80.0, 70–99)	81.8 ± 6.6 (83.00 68–97)	0.074
Male	79.8 ± 6.5 (79.0, 68–98)	79.5 ± 6.5 (78.7, 70–98)	81.6 ± 6.5 (83.2 68–97)	
Female	81.6 ± 6.5; (81.6, 69–99)	81.5 ± 6.5 (81.4, 70–99)	81.9 ± 6.7 (82.7 69–96)	
Sex (n, %)				0.032
Male	237 (50.6%)	208 (52.8%)	29 (39.2%)	
Female	231 (49.4%)	186 (47.2%)	45 (60.8%)	
Overall survival				< 0.001*
Survived (n, %)	193 (41.2%)	145 (36.8%)	48 (64.8%)	
Male	92 (47.7%)	76 (52.4%)	16 (33.3%)	
Female	101 (52.3%)	69 (47.6%)	32 (66.6%)	
Died in the hospital (n, %)	124 (26.5%)	121 (30.7%)	3 (4.1%)	
Male	66 (53.2%)	65 (53.7%)	3 (100%)	
Female	58 (46.8%)	56 (46.3%)	0 (80.0%)	
Died after discharge (n, %)	85 (18.2%)	69 (17.5%)	16 (21.6%)	
Male	50 (58.8%)	41 (59.4%)	9 (56.3%)	
Female	35 (41.2%)	28 (40.6%)	7 (43.7%)	
Unknown	66 (14.1%)	59 (15%)	7 (9.5%)	
Duration of treatment at the University (d)	13.3 ± 13.6 (9.2, 0.1–108)	13.23 ± 13.83 (9.36, 0.07-107.96)	13.72 ± 12.53 (9.08, 0.08–66.9)	0.441
Intensive care				0.932
Yes	135 (28.8%)	114 (28.9%)	21 (28.4%)	
No	333 (71.2%)	280 (71.1%)	53 (71.6%)	

*Difference is significant.

**Table 2:** Geriatric assessment of the patients with early complex geriatric rehabilitation (*n* = 74).

Assessment	Total (MW ± SD; Median, Min-Max, *n*)	*p*-value
Barthel-index at time of admission	39.0 ± 23.7 (42, 0–90, 69)	
Barthel-index at time of discharge	53.3 ± 31.3 (60, 0–95, 63)	0.007*
In-patient stay at geriatric department (d)	18.6 ± 10.3 (16.8, 1–55, 74)	
TUG at time of admission (sec)	22.3 ± 13.1 (19.8, 0–61, 16)	
TUG at time of discharge (sec)	21.0 ± 12.8 (18.3, 10–75, 30)	0.009*
MMST at time of admission	20.1 ± 7.7 (22.7, 0–30, 61)	
GDS at time of admission	3.0 ± 2.7 (2.4, 0–13, 59)	

*TUG* timed-up-and-go test, *MMST* Mini-Mental-State-Test, *GDS* geriatric depression scale.

*Difference is significant.

### Clinical situation at 12 months after discharge from the hospital

In our study 193 persons, who fulfilled the criteria for geriatric patients, were alive at the 12 month evaluation. Of these, 51 agreed to participate in the study (Fig. 1). We divided these 51 patients (mean age: 80.2 ± 6.4 y; 26 females) into two groups: one group who had received “early complex geriatric rehabilitation” at the Department of Geriatrics after their discharge from the university hospital (*n* = 16; mean age: 80.9 ± 7.6 years; 11 females), and the second group who had not received this additional treatment (*n* = 35; mean age: 79.8 ± 5.8 years; 15 females) (Table 3).

**Table 3:** Patients, one year after discharge with and without early complex geriatric rehabilitation (*n* = 51).

Variable	Whole cohort (*n* = 51; $\overline {{\text{x}}}$±SD; median, range, n)	Patients no early complex geriatric rehabilitation (*n* = 35; $\overline {{\text{x}}}$±SD; median, range, n)	Patients with early complex geriatric rehabilitation (*n* = 16; $\overline {{\text{x}}}$±SD; median, range, n)	*p*-value
Age	80.2 ± 6.4 (79.5, 69–98, 51)	79.8 ± 5.8 (78.7, 71–98, 35)	80.9 ± 7.6 (81.5, 69–93, 16)	*p* = 0.470
Male	79.0 ± 6.7 (77.7, 69–98, 25)	79.4 ± 6.8 (77.8 71–98)	77.8 ± 6.5 (77.0, 69–87, 5)	
Female	81.2 ± 6.1 (82.0, 71–93, 26)	80.4 ± 4.5 (81.0, 71–87)	82.4 ± 7.9 (86.0, 71–93, 11)	
Sex (n, %)				*p* = 0.086
Male	25 (49.0%)	20 (57.1%)	5 (31.3%)	
Female	26 (51.0%)	15 (42.9%)	11 (68.7%)	
Current medication (n, %)				*p* = 0.208
1–4 medications	21 (41.17%)	12 (34.28%)	9 (56.25%)	
5–9 medications	18 (35.29%)	12 (34.28%)	6 (37.50%)	
Over 10 medications	7 (13.72%)	6 (17.14%)	1 (6.25%)	
No data	5 (9.80%)	5 (14.28%)	0 (0%)	
Care level (n, %)				*p* = 0.481
No care level	26 (51.0%)	20 (57.1%)	6 (37.5%)	
Care level 1	2 (3.9%)	1 (2.9%)	1 (6.3%)	
Care level 2	9 (17.6%)	5 (14.3%)	4 (25%)	
Care level 3	8 (15.7%)	5 (14.3%)	3 (18.8%)	
Care level 4	5 (9.8%)	4 (11.4%)	1 (6.3%)	
Care level 5	1 (2.0%)		1 (6.3%)	
Duration of treatment (d)	27.3 ± 28.3 (20.0, 0-150, 45)	21.9 ± 26.2 (14.8, 0-150, 35)	46.1 ± 28.4 (40.5, 14–90, 10)	*p* = 0.004*
Actions during treatment (n, %)				*p* = 0.843
Medicative treatment	42, (82.4%)	28, (80.0%)	14, (87.5%)	
Isolation	47, (92.2)	34, (97.1%)	13, (81.3%)	
Ventilation	11, (21.6%)	7, (20%)	4, (25.1%)	
Psychological care	2, (3.9%)	1, (2.9%)	1, (6.3%)	
Intensive care (n, %)	20, (39.2%)	12, (34.3%)	8, (50%)	*p* = 0.286

*Difference is significant.

Following hospital treatment patients were dismissed either to the respite care, to their homes or residency care. No difference could be found among the two groups. The majority of persons lived at home (*n* = 41, 80.4%) with ten persons (19.6%) residing in nursing homes (Table 4). In the group without early complex geriatric rehabilitation, more persons lived at home (*n* = 30, 85.7%) than in the group with early complex geriatric rehabilitation (11, 68.7%).

**Table 4:** Care of patients with and without early complex geriatric rehabilitation after one year (*n* = 51).

Variable	Whole cohort (*n* = 51; %)	Patients no early complex geriatric rehabilitation (*n* = 35; %)	Patients with early complex geriatric rehabilitation (*n* = 16; %)	*p*-value
Main caretaker (n, %)				0.134
No care needed	25 (49.0%)	20 (57.1%)	5 (31.3%)	
Child	7 (13.7%)	3 (8.6%)	4 (25%)	
Partner/spouse	6 (11.8%)	5 (14.3%)	1 (6.3%)	
Other (nursing home, assistant living)	13 (25.5%)	7 (20.0%)	6 (37.5%)	
Housing situation (n, %)				0.157
Own home	41 (80.4%)	30 (85.7%)	11 (68.7%)	
Nursing home	10 (19.6%)	5 (14.3%)	5 (31.3%)	
Care situation (N, %)				0.162
Family care	11 (21.6%)	6 (17.1%)	5 (31.3%)	
Outpatient care	32 (62.7%)	25 (71.5%)	7 (43.8%)	
Stationary care	8 (15.7%)	4 (11.4%)	4 (25%)	

*Difference is significant.

When evaluating the care provision of the persons one year after discharge from the hospital in the follow-up cohort, 25 (49%) did not require any additional care. Moreover, in Germany there is a distinction between level of care and financial support (“Pflegegrad”, PG) based on the level of assistance needed, ranging from I-V. (no PG: 26 persons (51%); PG-I: 2 (3,9%); PG-II: 9 (17,6%); PG-III: 8 (15,7%); PG-IV: 5 (9,8%); PG-V: 1 (2%)). The level of care also differed between the two subgroups. In the group without early complex geriatric rehabilitation, more persons had no PG compared to the group with early complex geriatric rehabilitation (20, 57.1%; 6, 37.5%) (Table 3).

To evaluate the basic activities of daily living, the Barthel Index at 12 months was assessed resulting in a mean of 84.8 ± 21.9 points. Interestingly, the mean value was higher in the cohort without early complex geriatric rehabilitation than in the group who had received early complex geriatric rehabilitation (87.9 ± 17.5 vs. 78.1 ± 29.1 points). However, it showed no significant difference. This index is influenced by several different factors, and in this case, age plays a considerable role (Tables 5, 6 and 7) The Barthel Index in this group of persons that received complex treatment rose from 39.0 ± 23.7 at the time of admission to the geriatric hospital to 53.3 ± 31.3 at the time of discharge (*p* = 0.030) and to 78.1 ± 29.1 (*p* = 0.335) at the time of data collection one year after discharge. Unfortunately, no data was collected for the group of persons, that did not receive complex geriatric treatment, at the time of in-patient care.

**Table 5:** Outcomes of patients with and without early complex geriatric rehabilitation after one year (*n* = 51).

Variable	Whole cohort (*n* = 51; $\overline {{\text{x}}}$±SD; median, range, n)	Patients no early complex geriatric rehabilitation (*n* = 35; $\overline {{\text{x}}}$±SD; median, range, n)	Patients with early complex geriatric rehabilitation (*n* = 16; $\overline {{\text{x}}}$±SD; median, range, n)	*p*-value
Barthel index	84.8 ± 21.9 (95.9, 10–100, 51)	87.9 ± 17.5 (96.6, 45–100, 35)	78.1 ± 29.1 (92.5, 10–100, 16)	0.14
TUG (min)	19.2 ± 19.7 (11.75, 6-105, 37)	16.8 ± 13.2 (11.0, 6–65, 29)	25.3 ± 34.5 (12.0 8-105, 8)	0.14
MMST	26.9 ± 3.6 (28.2, 13–30, 50)	27.6 ± 2.7 (28.5, 21–30, 35)	25.4 ± 5.0 (27, 13–30, 15)	0.05
AES	33.5 ± 11.7 (32.4, 18–60, 51)	32.42 ± 10.8 (32.3, 18–60, 35)	35.7 ± 13.7 (32.5, 18–59, 16)	0.35
GDS	4.2 ± 3.5 (3.3, 0–13, 50)	3.6 ± 3.1 (3, 0–12, 35)	5.7 ± 4.1 (4, 0–13, 15)	0.06
EQ-5D-5 L VAS	60.4 ± 19.9 (64.2, 0–95)	65.4 ± 15.5 (67.7, 30–95)	49.4 ± 24.0 (51.6, 0–90)	0.006*

*TUG* timed-up-and-go test, *MMST* Mini-Mental-State-Test, *AES* apathy evaluation scale, *GDS* geriatric depression scale, *EQ-5D-5L VAS* EuroQuol Visual Analog Scale.

*Difference is significant.

**Table 6:** Barthel-index mean change between discharge and admission of the patients with early complex geriatric rehabilitation (*n* = 74) among different age groups.

Age	Total Mean change between discharge and admission	*p*-value
70–75 years	15.8	0.031*
76–80 years	32.5	0.125
81–85 years	12.9	0.043*
86–90 years	22.6	0.000*
91–95 years	10	0.029*
> 95 years	8.3	0.750

*Difference is significant at *p* < 0.05.

**Table 7:** Barthel-Index of the patients with and without early complex geriatric rehabilitation (*n* = 51) after 1 year among different age groups.

Variable	Whole cohort (*n* = 51; $\overline {{\text{x}}}$±SD; median, range)	Patients no complex geriatric rehabilitation (*n* = 35; $\overline {{\text{x}}}$±SD; median, range)	Patients with complex geriatric rehabilitation (*n* = 16; $\overline {{\text{x}}}$±SD; median, range)	*p*-value
Age				
70–75 years	90.4 ± 16.8 (100, 55–100)	91.4 ± 14.6 (100, 70–100)	88.7 ± 22.5 (100, 55–100)	0.999
76–80 years	91.7 ± 18.3 (100, 35–100)	96.4 ± 10.8 (100, 60–100)	70.0 ± 32.7 (75, 35–100)	0.044*
81–85 years	75.0 ± 27.5 (80, 10–100)	80.0 ± 18.3 (80, 50–100)	52.5 ± 60.1 (52.5, 10–95)	0.527
86–90 years	86.1 ± 16.1 (95, 55–100)	76.7 ± 22.5 (75, 55–100)	90.8 ± 11.5 (95, 70–100)	0.476
91–95 years	67.5 ± 45.9 (67.5, 35–100)	100 ±. (100, 100–100)	35.0 ±. (35, 35–35)	
> 95 years	45.0 ±. (45, 45–45)	45 ±. (45, 45–45)	0	

*Difference is significant at *p* < 0.05.

The mean TUG time was 19.2 ± 19.7 s. The cohort without early complex geriatric rehabilitation had a approximately 33.6% faster TUG time than the cohort with early complex geriatric rehabilitation (16.8 ± 13.2 vs. 25.3 ± 34.5 s). This potentially due to the latter cohort, more people were living in a nursing home and were sitting in a wheelchair or were confined to bed, where they were not able to participate in the TUG test. Moreover, they had more comorbidities. However, it should be noted that the results are not significant.

Drug consumption at 12 months was also different between the two groups. We divided the number of medications to be taken into groups: 1–4 medications, 5–9 medications, and over 10 medications. The group with early complex geriatric rehabilitation had to take less often over 10 medications but instead more 1–4 medications compared to the group without early complex geriatric rehabilitation (with: 1–4: 9, 56.25%; 5–9, 6, 37.5%; over 10, 1, 6.25%) (without: 1–4: 12, 34.28%; 5–9, 12, 34.28%; over 10, 6, 17.14%) (no significance) (Table 3).

To score the emotional feelings of the patients, we used the GDS. The whole study group had a mean value of 4.2 ± 3.5 points. The group with early complex geriatric rehabilitation had a around 58.33% higher score (5.7 ± 4.1 points) than the group without geriatric care (3.6 ± 3.1 points) (Table 5) (not significant). In terms of apathy or lack of motivation, the mean AES score was 33.5 ± 11.7 points, with both groups remaining below the cut-off value. In the group with geriatric care (35.7 ± 13.7 points), higher values were found compared to the group without early complex geriatric rehabilitation (32.4 ± 10.8 points) (Table 5) (no significance).

Finally, we evaluated health status using the EQ-5D-5 L-VAS (EuroQol visual analogue scale). The group achieved a mean value of 60.4 ± 19.9 points. The patients in the group without early complex geriatric rehabilitation scored higher (65.4 ± 15.5 points) than the patients with early complex geriatric rehabilitation (49.4 ± 24 points). The group without early complex geriatric rehabilitation scored approximately 24.5% lower, compared to the group without complex geriatric treatment. (Table 5) (*p* = 0.006).

## Discussion

The aim of this study was to investigate the journey of geriatric individuals with COVID-19 during the pandemic retrospectively and their outcomes one year later prospectively in a German academic setting. There are a number of interesting results: First, in the retrospective part of our study, the mortality rate at the time of discharge in our cohort was slightly lower than that in a comparable German study (26.5% vs. 27.9%) with similar mean ages (80.7 vs. 80.6 years), which, however, included only 168 patients in their study^36^. These German mortality rates, however, are considerably higher when compared to the numbers published in other European countries. For instance, in Italy until April 2020, 19.7% of patients over 80 years died due to or related to COVID-19^11^. In France, 18% of patients with a mean age of 85.5 years died^37^.

Second, it is worth noting that nearly half of the geriatric patients had succumbed to the disease one year later. This represents a substantial increase in excess mortality within these age groups. Unfortunately, our study did not explore the prevailing causes in more detail. As suggested by various studies, age is the most prominent risk factor for severe coronavirus progression and adverse health outcomes^38–40^. Our results substantiate that there is a relationship between age, infection, and mortality rate, especially following the acute infection condition. In our cohort, 28.8% of patients had severe COVID-19 and needed treatment in an intensive care unit, which is slightly higher than the number of patients in the cohort of Dörr et al. (25%). Other studies from the USA or the UK reported ICU admissions of 23.6% and 17%, with median ages of 63 and 73 years, respectively^41,42^. This indicates a considerable influence of the factor age concerning the course of disease.

Third, patients who received early complex geriatric rehabilitation had a significantly higher chance for long-term survival than those who did not. Although the number of subjects included in this study was low, the results provide evidence that specialized geriatric care following acute infection of COVID-19 may contribute to increased long-term survival for these individuals. To the best of our knowledge, we could not identify articles to compare our results, as “early complex geriatric rehabilitation” was mostly suggested for surgical patients^43^. Therefore, elaborated studies are urgently needed to evaluate the most appropriate care for geriatric patients during a pandemic situation as “the next pandemic is behind the next corner”.

Fourth, the duration of inpatient stay in our cohort was 13.3 days, which correlates to the cohort of Dörr et al. (12 days)^36^. Notably, this is much longer than the mean duration of in-patient stay in German hospitals (2021; 8.8 days)^44^ resulting in considerable higher costs for the respective health care system. Furthermore, there are no relevant differences regarding the length of stay, whether intensive care was necessary or not and the age of the patients.

In the prospective part of this study, in all the geriatric assessments (Barthel, TUG, MMST, AES, GDS), the patients who also received early complex geriatric rehabilitation performed worse compared to the “control group”. However, these findings need to be interpreted cautiously, as the patients who received early complex geriatric rehabilitation were generally older and suffered from more comorbidities. When assessing the data, it was evident that the circumstances of the two cohorts was markedly different. The patients who did not receive the early complex geriatric rehabilitation presented a better general overall condition. They were all able to travel to the university hospital for examinations, as in the cohort with early complex geriatric rehabilitation, we had to conduct more home visits due to their reduced mobility. Another fact is that only these patients who needed early complex geriatric rehabilitation were transferred to the Geriatric Department for this specialized care. Those who did not require this treatment were discharged home, indicating that they were in a better condition compared to the other group.

Despite a detailed study design, the study may carry several limitations. First, we focused only on patients who were admitted to the hospital; thus, our data cannot be generalized to the whole population of COVID-19-infected persons aged over 70 years. Second, a number of patients were not available for the 12-month follow-up, which may introduce bias into the conclusions drawn. With only 51 participants in the prospective part, the sample size was small, interfering with the statistical power of the results. Third, the interviews were performed by three interviewers; despite the use of standardized assessments and adequate training, bias cannot be excluded. Forth, despite all efforts, during the high season of the COVID-19 pandemic there was no clear standardization yet, on which patients should be transferred to the Department of geriatrics, and which patients should not. Finally, the first part of the study included a retrospective data analysis, the significance of which is limited and can only be evaluated to a certain degree. Retrospective studies, however, still play a role in the understanding and interpretation of geriatric measurements and studies and may serve as a basis for future research.

## Conclusion

SARS-CoV-2 and other viruses are a widely threat, particularly for elderly people. The outcomes of COVID-19 can vary, but the death rates are tendentially higher than those of younger people. “Early complex geriatric rehabilitation” had a slightly positive effect on death rates but did not show improved outcomes in terms of geriatric assessments. More studies are needed to identify the most adequate treatment during a pandemic situation for a geriatric population.

## Data Availability

Availability of data and materials: on reasonable request all data and materials can be provided by the corresponding author.
